# Regression modeling of the North East Atlantic Spring Bloom suggests previously unrecognized biological roles for V and Mo

**DOI:** 10.3389/fmicb.2013.00045

**Published:** 2013-03-08

**Authors:** Nick J. Klein, A. J. Beck, D. A. Hutchins, S. A. Sañudo-Wilhelmy

**Affiliations:** ^1^Department of Earth Sciences, University of Southern CaliforniaLos Angeles, CA, USA; ^2^Department of Physical Sciences, Virginia Institute of Marine SciencesGloucester Point, VA, USA; ^3^Department of Biological Sciences, University of Southern CaliforniaLos Angeles, CA, USA

**Keywords:** trace nutrients, North Atlantic Spring Bloom, B-vitamins, vanadium, molybdenum

## Abstract

In order to identify the biogeochemical parameters controlling pCO_2_, total chlorophyll a, and dimethyl sulfide (DMS) concentrations during the North East Atlantic Spring Bloom (NASB), we used previously unpublished particulate and dissolved elemental concentrations to construct several linear regression models; first by hypothesis-testing, and then with exhaustive stepwise linear regression followed by leave-one-out cross-validation. The field data was obtained along a latitudinal transect from the Azores Islands to the North Atlantic, and best-fit models (determined by lowest predictive error) of up to three variables are presented. Total chlorophyll a is predicted best by biomass (POC, PON) parameters and by pigments characteristic of picophytoplankton for the southern section of the sampling transect (from the Azores to the Rockhall-Hatton Plateau) and coccolithophores in the northern portion (from the Rockhall-Hatton Plateau to the Denmark Strait). Both the pCO_2_ and DMS models included variables traditionally associated with the development of the NASB such as mixed-layer depth and with Fe, Si, and P-deplete conditions (dissolved Fe, dissolved and biogenic silica, dissolved PO^3−^_4_). However, the regressions for pCO_2_ and DMS also include intracellular V and Mo concentrations, respectively. Mo is involved in DMS production as a cofactor in dimethylsulfoxide reductase. No significant biological role for V has yet been determined, although intracellular V is significantly correlated (*p*-value <0.05) with biogenic silica (*R*^2^ = 0.72) and total chlorophyll a (*R*^2^ = 0.49) while the same is not true for its biogeochemical analogue Mo, suggesting active uptake of V by phytoplankton. Our statistical analysis suggests these two lesser-studied metals may play more important roles in bloom dynamics than previously thought, and highlights a need for studies focused on determining their potential biological requirements and cell quotas.

## Introduction

The North East Atlantic Spring Bloom (NASB) is a large annual phytoplankton bloom event triggered by a decrease in mixed-layer depth in March or April. It is typically characterized by early domination of diatoms, depletion of dissolved Si, and later succession by coccolithophores and other non-silicifying organisms (Sieracki et al., 1993). The dynamics of the NASB strongly influence the partial pressure of carbon dioxide (pCO_2_) in the region (Ducklow and Harris, 1993). The bloom is of particular interest in light of global climate change, owing to its status as a significant sink for anthropogenic CO_2_ (Gruber, 1996).

The NASB 2005 program set as its goals to describe the phytoplankton community structure during the late stages of the NASB and determine relative contributions of the major phytoplankton taxa (e.g., diatoms and coccolithophores) in export of carbon and biominerals (LeBlanc et al., 2009). The NASB 2005 cruise yielded a large amount of data, including a broad spectrum of phytoplankton pigments, atmospheric CO_2_, dimethyl sulfide (DMS), and trace metal and B-vitamins (B_12_ and B_1_) concentration data. We present previously unpublished dissolved and P-standardized particulate trace metal data, which are scarce in the literature for that geographical region (Kuss and Kremling, 1999). This publication aims to utilize the trace metal and B-vitamin data in combination with pigment and other environmental data to more fully describe nutrient limitation conditions observed during the 2005 NASB cruise, as well as to employ correlative statistical methods to produce predictive models describing any relationships between pCO_2_, chlorophyll a, and DMS with the wealth of other variables in the dataset. The three variables were selected to explore the relationship between primary production (represented by chlorophyll a) and production of the climactically important gases CO_2_ and DMS.

Due to the unexpected enrichment of the lesser-studied trace metal nutrients Mo and V in recent phytoplankton metal studies (Tovar-Sanchez and Sañudo-Wilhelmy, 2011; Nuester et al., 2012), special consideration of the potential roles and importance of these elements is given. Mo and V are the two most abundant transition metals in seawater, with typical average concentrations around 100 nmol L^−1^ (Collier, 1985) and 35 nmol L^−1^ (Dupont et al., 1991). Mo plays important biological roles, particularly in the nitrogen cycle, where it is a metal cofactor in nitrogenase and other enzymes involved in N-fixation and incorporation (Kisker et al., 1997). Mo is also the metal cofactor in dimethylsulfoxide reductase (Schindelin et al., 1996), an enzyme central to production of the modeled gas DMS. The only known biological roles for V in relation to plankton biology is as the metal cofactor of uncommon V-nitrogenases and in V-haloperoxidases (Crans et al., 2004).

## Area of study

**Figure 1:**
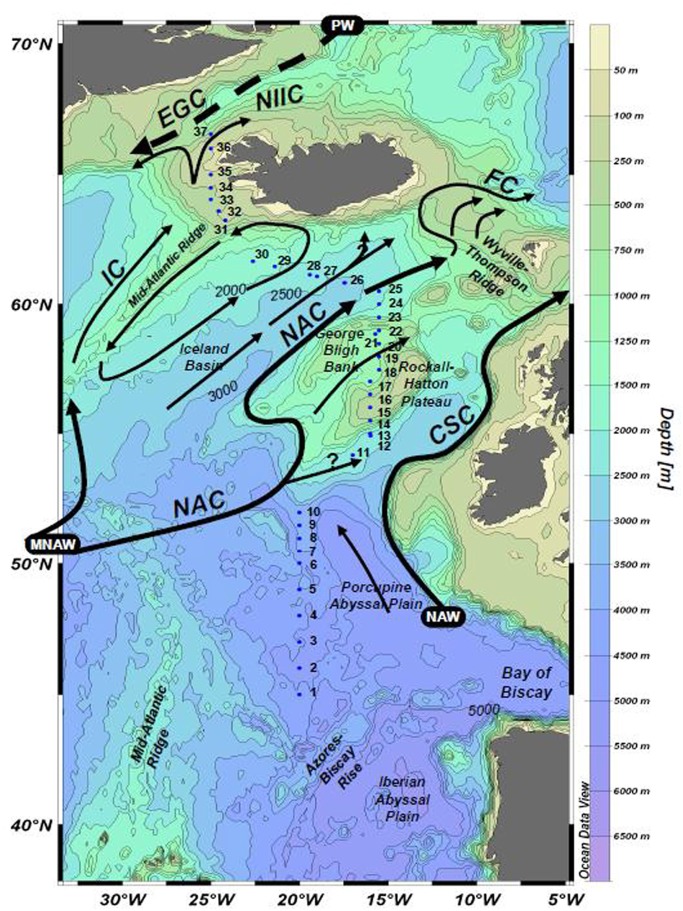
**Sampling locations along the NASB transect, shown with major surface currents.** Surface sampling stations are numbered 1–37. Figure from LeBlanc et al. (2009).

Sampling was conducted from 6 June to 3 July 2005 aboard the R/V Seaward Johnson II along a south–north transect of the northeast Atlantic Ocean (Figure 1), generally following the 20°W meridian. Real-time satellite data was monitored during the cruise, and the route adjusted slightly to sample areas where satellite data indicated coccolithophore blooms.

## Methods

Near-surface seawater (5–10 m depth) was pumped onboard using an acid-washed all-Teflon trace-metal clean pumping system (Osmonics Bruiser) extended away from the ship on a boom. Water was pumped directly into a trace metal clean van and filtered through a 0.22 μm acid-washed polypropylene capsule filter directly into 1 L acid-washed LDPE bottles. Dissolved trace metal samples were acidified to pH <2 with 6 N quartz-distilled HCl (Optima-grade) and preconcentrated following Bruland et al. (1985).

Particulate samples for metals determination was filtered onto duplicate acid-washed polycarbonate filter membranes (0.2 μm pore size) from between 0.13 and 4 L of seawater, depending on plankton abundance. For total metals content, particulates collected on one of the filters was rinsed with Chelex-cleaned trace metal-free seawater. For P-standardized particulate metal concentrations, biomass on the second filter was washed to remove surface-adsorbed metals using 10 mL of oxalate reagent (Tovar-Sanchez et al., 2003), although the reagent was not cleaned prior to use. Instead, biomass was rinsed following the oxalate wash with 5 aliquots of 10 mL Chelex-cleaned trace metal-free seawater similar to Tang and Morel (2006). To monitor the rinse efficiency and confirm that there was no contamination from the oxalate reagent, a blank filter was subjected to the oxalate wash and rinse procedure with every sample (*n* = 36). Phytoplankton biomass was digested with 2 mL aqua regia and 50 μL HF (all acids Optima-grade). The digests were evaporated to dryness, and the residue taken up in 2 mL of 1 N Optima-grade HNO_3_. Dissolved trace metal extracts and filter digests were analyzed by high resolution inductively-coupled plasma mass spectrometry (ICPMS; Thermo-Fisher Element 2) using indium as an internal standard.

The ancillary dataset was compiled from surface transect data (depth = 10 m) presented in LeBlanc et al. (2009) for a total of 51 variables across 27 surface transect stations.

Dissolved trace metal and nutrient data were compared to published literature stoichiometry to assess potential limitation. All statistical work was performed in the R statistical analysis program (R Development Core Team, 2010). Shorthand abbreviations (e.g. DIC for dissolved inorganic carbon) for each variable are used in the figures presented here, and a key for their interpretation may be found in Appendix.

Missing values (17% of 1404 total) were estimated using nearest-neighbor imputation (Hastie et al., 2010) and the data scaled and centered. This imputation method is not regression-based and does not produce significant bias or smoothing of the data (Chen and Shao, 2000). Hypothesis-driven regression models were constructed from variables of interest identified using existing literature (e.g., mixed-layer depth as a trigger for the NASB) and from the nutrient stoichiometry analyses. Following hypothesis-driven analysis, an exhaustive stepwise linear regression algorithm (Lumley, 2009) was employed and statistically significant regressions of up to three variables were retained for further consideration. As stepwise linear regression amounts to data mining and introduces the risk of Type III statistical errors (formulating hypotheses suggested from the data), leave-one-out cross-validation was performed to aid in selection of linear regression models better reflective of real trends and not data noise (Canty and Ripley, 2010).

## Results and discussion

**Figure 2:**
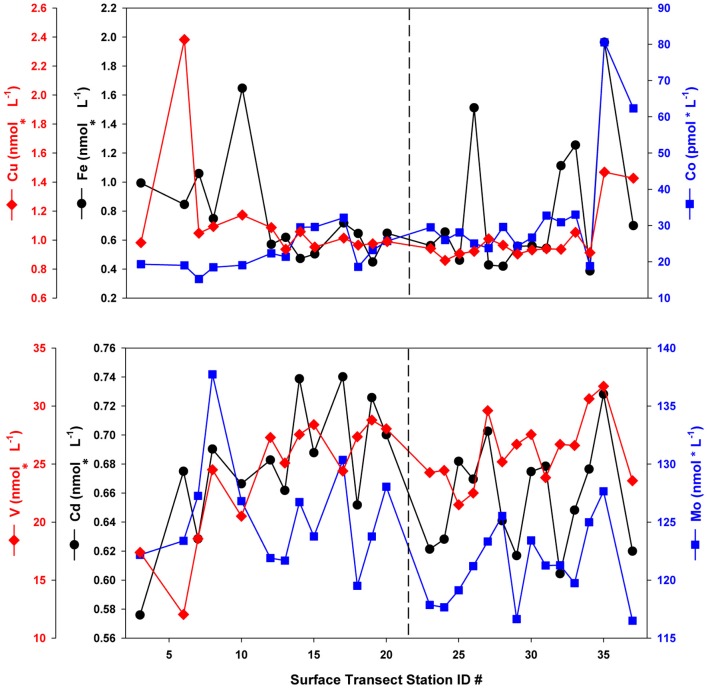
**Dissolved trace metal concentrations along the NASB transect (depth = 10 m).** Vertical dashed line separates the Southern (left panel, stations 1–23) from Northern transect (right panel, stations 24–37) sections.

**Figure 3:**
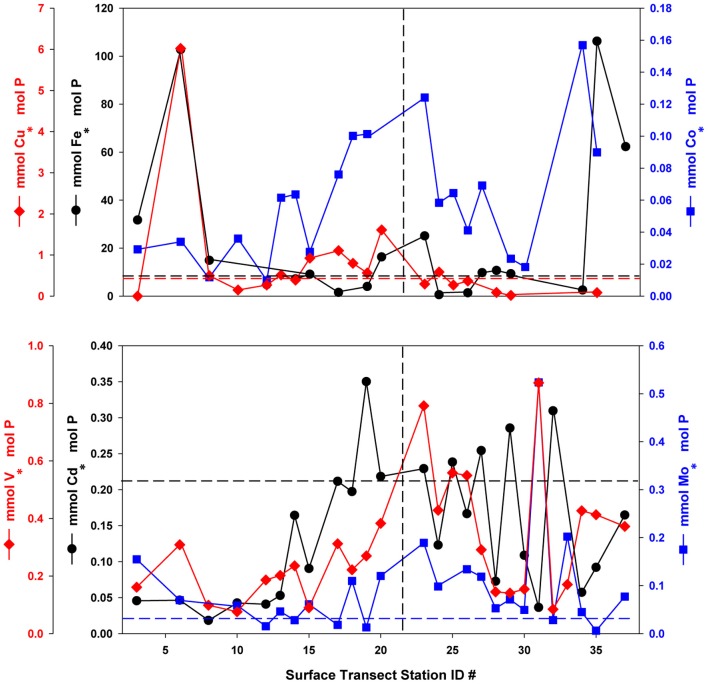
**Oxalate-washed P-standardized particulate trace metals along the NASB transect (depth = 10 m).** Vertical dashed line separates the Southern (left panel, stations 1–23) from Northern (right panel, stations 24–37) transect sections. Horizontal dashed lines are color-coded by element and correspond to median phytoplankton cellular quotas for that element from Ho et al. (2003). All particulate Co:P values were below the literature value of 0.19. No line is given for V:P due to a lack of laboratory culture data for comparison.

Prior to statistical analysis, the dataset was subdivided into two sections on the basis of their distinct hydrographic and biological regimes, a hypothesis confirmed by cluster analyses. A distinct surface salinity and temperature front separated what was subdivided as the southern transect from the northern transect section, and the two regions were observed to have different dominant phytoplankton taxa (see LeBlanc et al., 2009, Figure 2). This hypothesis was tested via application of k-means clustering (R Development Core Team, 2010) on the dataset, which produced two main clusters divided by the observed front, confirming the hypothesis. The following results and discussion consider the northern and southern transect sections separately, with the southern section stations (*n* = 13, station numbers 1–23) corresponding to the waters over the Porcupine Abyssal Plain and Rockhall-Hatton Plateau, while the northern section (*n* = 14, station numbers 24–37) represents those stations from waters overlying the Icelandic Basin and Shelf. Dissolved concentrations and oxalate-washed, P-standardized particulate metal content for bioactive trace metals considered in the nutrient limitation and stoichiometry (section “Nutrient Stoichiometry”) calculations (Fe, Cu, Co, Cd, Mo, and V) are presented in Figures 2, 3, respectively. A distinct concentration gradient was observed for dissolved Fe and Co, generally increasing northward (from 0.5 to 1 nmol L^−1^ and 20 to 35 pmol L^−1^ respectively), with a sharp peak observed in the Denmark Straight influenced by ice melt-waters (2 nmol L^−1^ Fe and 80 pmol L^−1^ Co) (Figure 2). Dissolved Mo and Cd ranged from 116 to 137 nmol L^−1^ and 0.58 to 0.74 nmol L^−1^, respectively, with neither element displaying a clear latitudinal trend. Dissolved V ranged from 12 to 32 nmol L^−1^, with values in the three southernmost stations appearing depleted relative to the remainder of the transect, where V varied between 20 and 30 nmol L^−1^ (Figure 2).

P-standardized particulate metal concentrations (Figure 3) were plotted with typical literature phytoplankton cellular quota values derived from laboratory culture experiments (Ho et al., 2003) with dashed lines for reference. Though these values are assumed to represent only the biological fraction for purposes of nutrient stoichiometry, the potential for a significant lithogenic contribution cannot be discounted. P-standardized particulate Fe concentrations ranged from less than 0.01 to 0.14 mmol · mol^−1^ P and were generally below or near the typical literature culture value of 7.5 mmol · mol^−1^ P, with stations at the extreme south and north of the transect being enriched by an order of magnitude. P-standardized particulate Cu concentrations ranged from 0.02 to 1.61 mmol · mol^−1^ P, and were generally below the literature value of 0.38 mmol · mol^−1^ P in the northern transect, excepting portions of the southern transect where they were enriched 2-fold. In contrast, observed P-standardized particulate Co concentrations (0.01–0.16 mmol · mol^−1^ P) were below the typical laboratory culture value of 0.19 mmol · mol^−1^ P at all stations. P-standardized particulate Cd levels (0.02–0.35 mmol · mol^−1^ P) are generally below or slightly above the literature value of 0.21 mmol · mol^−1^ P, while Mo (0.01–0.52 mmol · mol^−1^ P) is enriched above the culture value of 0.03 mmol · mol^−1^ P at most stations. Oxalate-washed particulate V:P ranged from 0.076 to 0.87 mmol · mol^−1^ P, but unfortunately no similar laboratory culture data exist with which to compare these values.

### Nutrient stoichiometry

To assess the potential for nutrient limitation and the relative importance of the various nutrient elements during the 2005 NASB cruise, the observed range and median value of dissolved and P-standardized particulate concentrations were compared to values derived from laboratory culture experiments (Brzezinski, 1985; Ho et al., 2003; Tang et al., 2010) and are presented in Table 1. Though there is significant variability between stations, dissolved inorganic N:P is near the Redfield stoichiometric value of 15 mol · mol^−1^ P for both transect sections with median ratios (Table 1) of 15 and 14 mol · mol^−1^ P for the southern and northern sections, respectively (transect values will be presented in the order of southern followed by northern from here onwards). Median particulate ratios of N:P are also near Redfield (Redfield, 1934), with values of 17 mol · mol^−1^ P for both sections of the transect (Table 1). In this treatment, the similarity of the measured dissolved and P-standardized particulate values to the laboratory culture data suggest that N is not depleted (relative to P) in either the dissolved or intracellular phase, and therefore is likely not limiting.

**Table 1:** **Comparison of the range and median values (in parentheses) of dissolved and oxalate-washed, particulate nutrients with literature values from laboratory culture experiments, standardized to P**.

	**Dissolved**		**Oxalate-washed P-standardized particulate**		**Laboratory culture**
	**Southern transect**	**Northern transect**	**Southern transect**	**Northern transect**	
N	14–21 (15)	10–36 (14)	9.6–20 (17)	13–25 (17)	5.4–38 (16)
Si	0.2–4.5 (1.9)	0.30–8.0 (1.8)	0.30–1.9 (1.6)	0.80–7.0 (3.7)	15
Fe	1.6–7.9 (2.5)	1.1–16 (2.6)	1.5–100 (9.1)	0.56–110 (9.8)	0.30–15 (7.5)
Cu	2.8–8.0 (4.9)	1.7–11 (3.9)	0.15–1.1 (0.52)	0.018–0.58 (0.27)	0.0060–1.4 (0.38)
Co	0.064–0.17 (0.093)	0.058–0.61 (0.11)	0.010–0.10 (0.036)	0.018–0.12 (0.060)	0.010–0.46 (0.19)
Cd	2.0–5.5 (3.8)	1.2–5.6 (2.5)	0.018–0.35 (0.072)	0.036–0.31 (0.16)	0.068–0.73 (0.21)
Mo	350–1000 (720)	220–980 (450)	0.013–0.15 (0.059)	0.006–0.20 (0.074)	0.0090–0.11 (0.033)
V	70–210 (99)	43–240 (94)	0.076–0.38 (0.20)	0.085–0.87 (0.39)	
B_1_	16–150 (37)	2.0–110 (20)			38–740 (150)
B_12_	1.2–9.8 (5.5)	0.72–17 (3.0)			0.050–500 (4.1)

N and Si are in units of mol · mol^−1^ P, trace metals in mmol · mol^−1^ P, and B-vitamins in nmol · mol^−1^ P, Trace metal and N values are from Ho et al. (2003), B-vitamins are from those complied in Tang et al. (2010), and Si values are from Brzezinski (1985).

Following the same logic, Cu, Cd, Mo, and vitamin B_12_ are enriched in the dissolved phase relative to observed P-standardized particulate and laboratory culture values (Table 1) and therefore unlikely to be limiting during the sampling period. Dissolved Cu:P (Table 1) is an order of magnitude greater than literature values with observed concentrations of 3.9 and 4.9 vs. 0.38 mmol · mol^−1^ P in laboratory culture (Ho et al., 2003), suggesting it is replete in the dissolved phase. Particulate Cu:P (Table 1) generally falls within the range of literature values, with median values of 0.52 and 0.27 mmol · mol^−1^ P bracketing the median literature value of 0.38 mmol · mol^−1^ P (Ho et al., 2003). Dissolved Cd:P (Table 1) is present in ratios of 3.8 and 2.5 mmol · mol^−1^ P, an order of magnitude greater than the laboratory culture stoichiometry value of 0.21 mmol · mol^−1^ P (Ho et al., 2003), and the range and median of Cd:P values fall within the lower range of culture values.

Mo is enriched in the dissolved phase (Table 1), with median values of 720 and 450 mmol · mol^−1^ P relative to 0.033 in culture (Ho et al., 2003), Median particulate Mo:P values (Table 1) of 0.059 and 0.074 fall in the middle of the laboratory culture concentration range of 0.0090 to 0.11 mmol · mol^−1^ P (Table 1). Dissolved V had observed median concentrations of 99 and 94 mmol · mol^−1^ P, while oxalate-washed particulate V:P was a median of 0.20 and 0.39 mmol · mol^−1^ P. There is a lack of similar laboratory algal culture V:P data for comparison as used for the other elements discussed here. However, V is the third most abundant of the metals measured in our samples (behind Fe and Cu), and was one to two orders of magnitude greater in relative abundance than the remaining three metals (Co, Cd, and Mo) at most stations (Table 1). In a recent study on field-sampled colonies of the nitrogen-fixing cyanobacterium *Trichodesmium*, Nuester et al. (2012) observed values of 13–63 mmol Fe · mol^−1^ P, which are similar to those reported here. However, in that study V:P was both threefold Fe:P and had the highest relative abundance of all measured metals. When compared to the Nuester et al. (2012) study, our data suggest that V content and abundance relative to other metals within algae may vary significantly.

**Figure 4:**
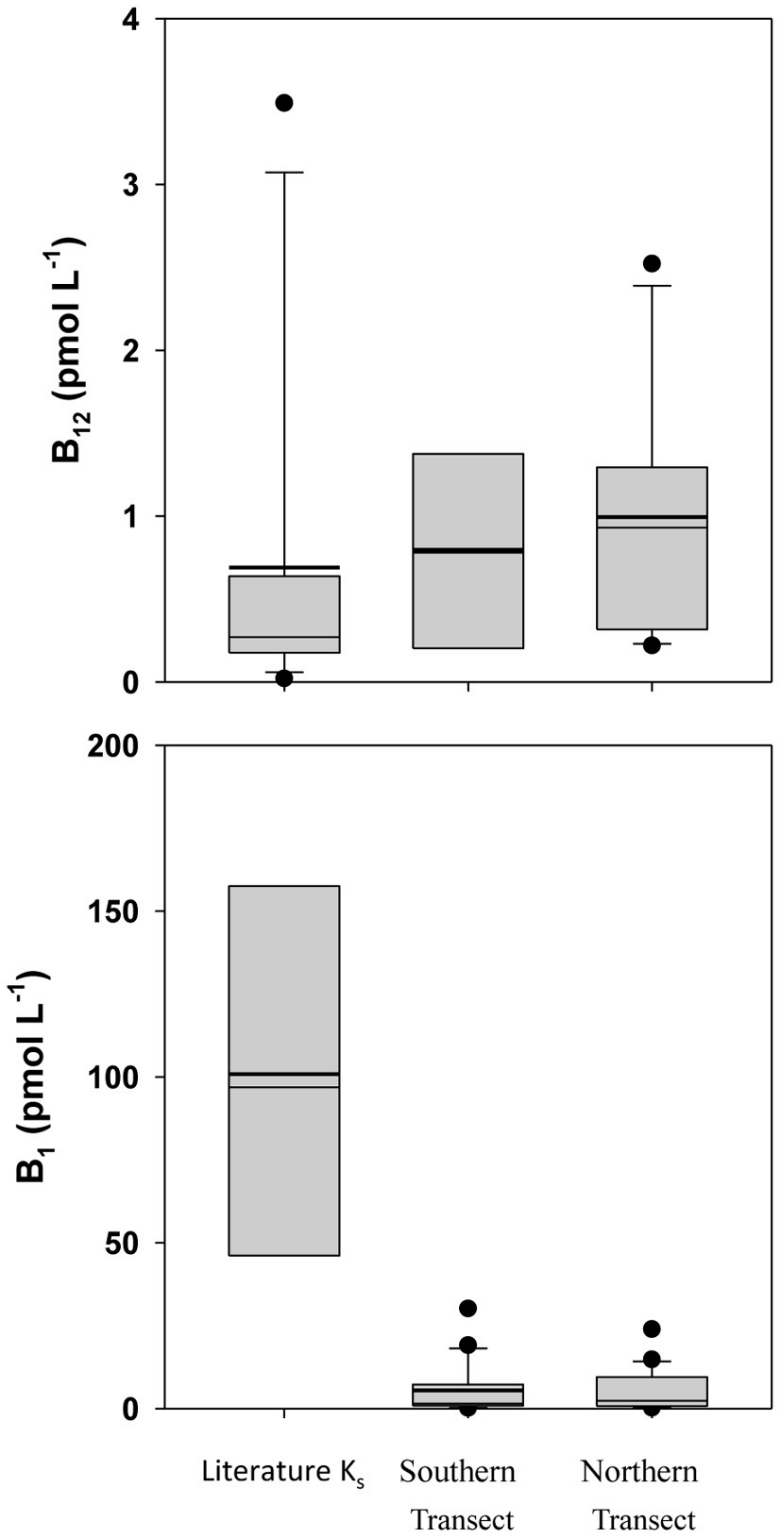
**Box-and-whisker plot Comparison of observed B-vitamin concentrations with literature Ks half-saturation constants for growth.** Dots represent outlier values (Taylor and Sullivan, 2008; Tang et al., 2010).

Literature data on B-vitamin requirements for phytoplankton is very limited, but on a stoichiometric basis, B_12_ would appear to be present in excess (Table 1), with median B_12_:P ratios (5.5 and 3.0 nmol · mol^−1^ P) similar to the median laboratory culture stoichiometric value of 4.1 nmol · mol^−1^ P (Tang et al., 2010). When observed B-vitamin concentrations are compared to literature K_s_ half-saturation constants for growth (Figure 4), B_12_ again appears replete while observed B_1_ concentrations are an order of magnitude lower than literature K_s_ values. As B_1_ is required for 49% of assayed dinoflagellate species, 15% of diatoms, and 83% of coccolithophores (Tang et al., 2010), it may be limiting to growth rates of those taxa and therefore selectively favor prototrophic species.

Fe, Si, Co, and B_1_ all exhibit dissolved nutrient:P ratios (Table 1) lower than observed P-standardized particulate and laboratory culture values (Table 1), suggesting these nutrients are potentially limiting or co-limiting on the NASB. Although the median oxalate-washed particulate Fe:P values of 9.1 and 9.8 mmol · mol^−1^ P are slightly above the median laboratory culture value of 7.5 (Ho et al., 2003), ratios of 2.5 and 2.6 mmol · mol^−1^ P in the dissolved phase appear significantly depleted. There is other evidence for Fe as a likely limiting or co-limiting element, as previous studies have demonstrated Fe limitation both during the development of the NASB (Moore, 2006) and during post-bloom conditions (Nielsdóttir et al., 2009), and Fe addition experiments during the 2005 NASB cruise stimulated chlorophyll a concentrations above control (LeBlanc et al., 2009).

Si:P is depleted well below the extended Redfield stoichiometry reported for diatoms (Brzezinski, 1985), with values of 1.9 and 1.8 mol · mol^−1^ P in the dissolved phase (Table 1) and oxalate-washed P-standardized particulate concentrations of 1.6 and 3.7 (Table 1) vs. 15 in the literature (Table 1), suggesting it would be limiting on those organisms (such as diatoms) which require Si. Co:P is depleted both in the dissolved (median values 0.093 and 0.11) and particulate phases (median of 0.036 and 0.060) relative to the median literature value from laboratory cultures of 0.19 nmol · mol^−1^ P (Ho et al., 2003). Despite being lower than the values observed in laboratory culture, oxalate-washed particulate Co:P in this study was several fold less than dissolved Co:P. The Co:P enrichment in the dissolved phase relative to depletion in the particulate phase suggests that a proportion of the dissolved Co is probably unavailable to the phytoplankton, which has been previously reported (Saito and Moffett, 2001). Dissolved vitamin B_1_, in addition to being present in quantities below reported K_s_ half-saturation constants for growth (Figure 4), falls on the low end of median laboratory culture stoichiometries (37 and 20 vs. 150 mmol · mol^−1^ P) and is thus a potentially limiting nutrient to B_1_ auxotrophs.

Overall, the comparison of nutrient stoichiometric ratios support the conclusions of LeBlanc et al. (2009) that the NASB at the time of sampling was in its late stages and had progressed beyond initial diatom dominance, which is reflected in the strong depletion of and potential limitation for diatom limitation by dissolved Si. The stoichiometry supports previous findings of mid- and post-bloom Fe limitation in the North Atlantic, and indicates that Co and vitamin B_1_ may also have the potential to have been limiting or co-limiting at the time of sampling.

### Linear regression modeling of pCO_2_, chlorophyll a, and DMS

Linear regression models for pCO_2_, chlorophyll a, and DMS were constructed first with hypothesis-testing based on potential nutrient limitation as discussed in section “Nutrient Stoichiometry” (dissolved Si, inorganic N, Fe, B_1_, and Co) and with mixed-layer depth, which is classically thought to trigger the NASB (Ducklow and Harris, 1993). Asterisked variable combinations in Tables 2, 3, and 4 (variable abbreviations defined in Appendix) denote statistically significant models constructed from this initial hypothesis-testing.

**Table 2:**
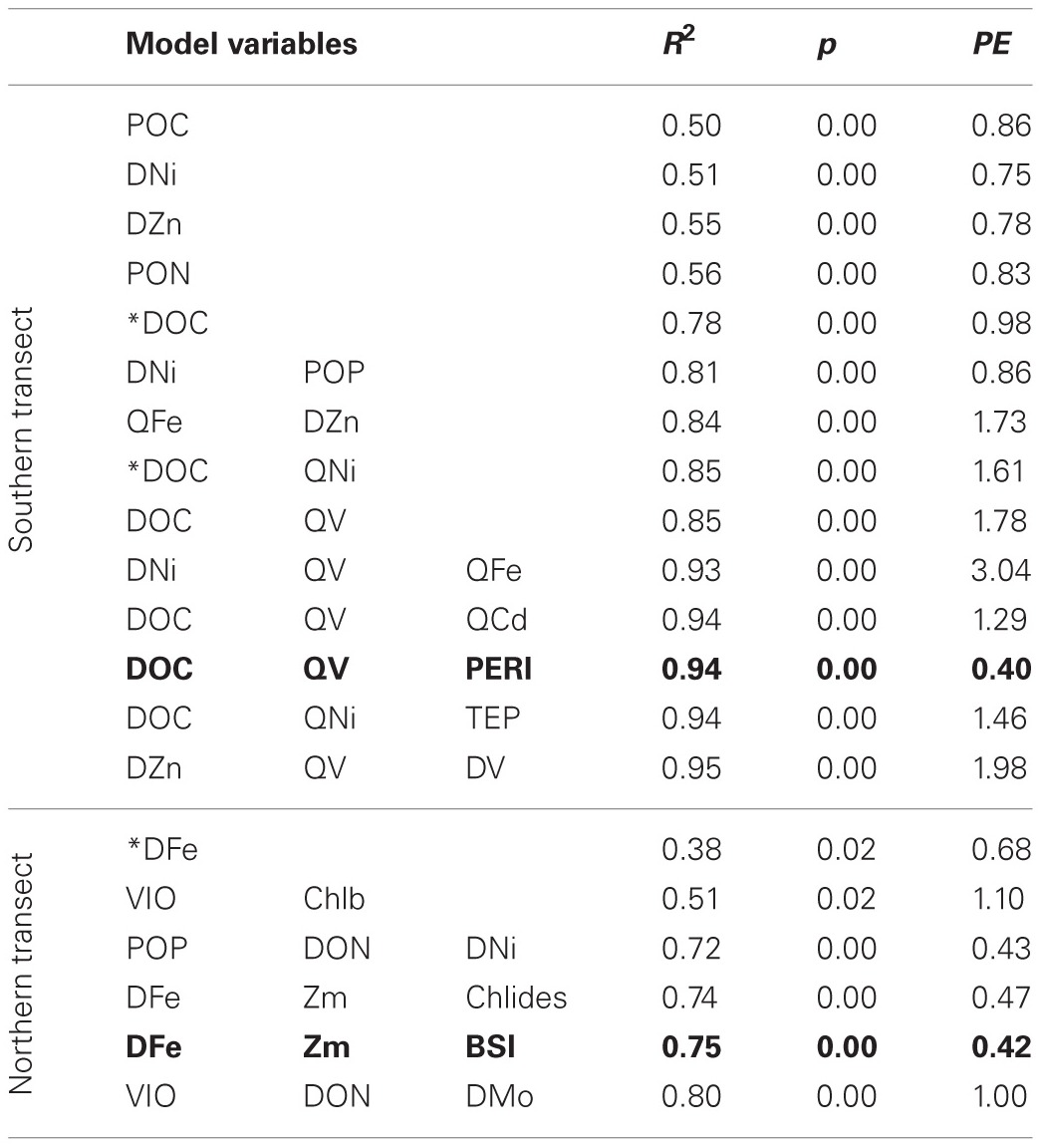
**Linear models and diagnostic statistics for pCO_2_, only statistically significant regressions for up to three variables are presented**.

Models with the lowest predictive error (PE), determined by leave-one-out cross validation, are bolded. Models produced from hypothesis-testing are marked with an asterisk.

**Table 3:**
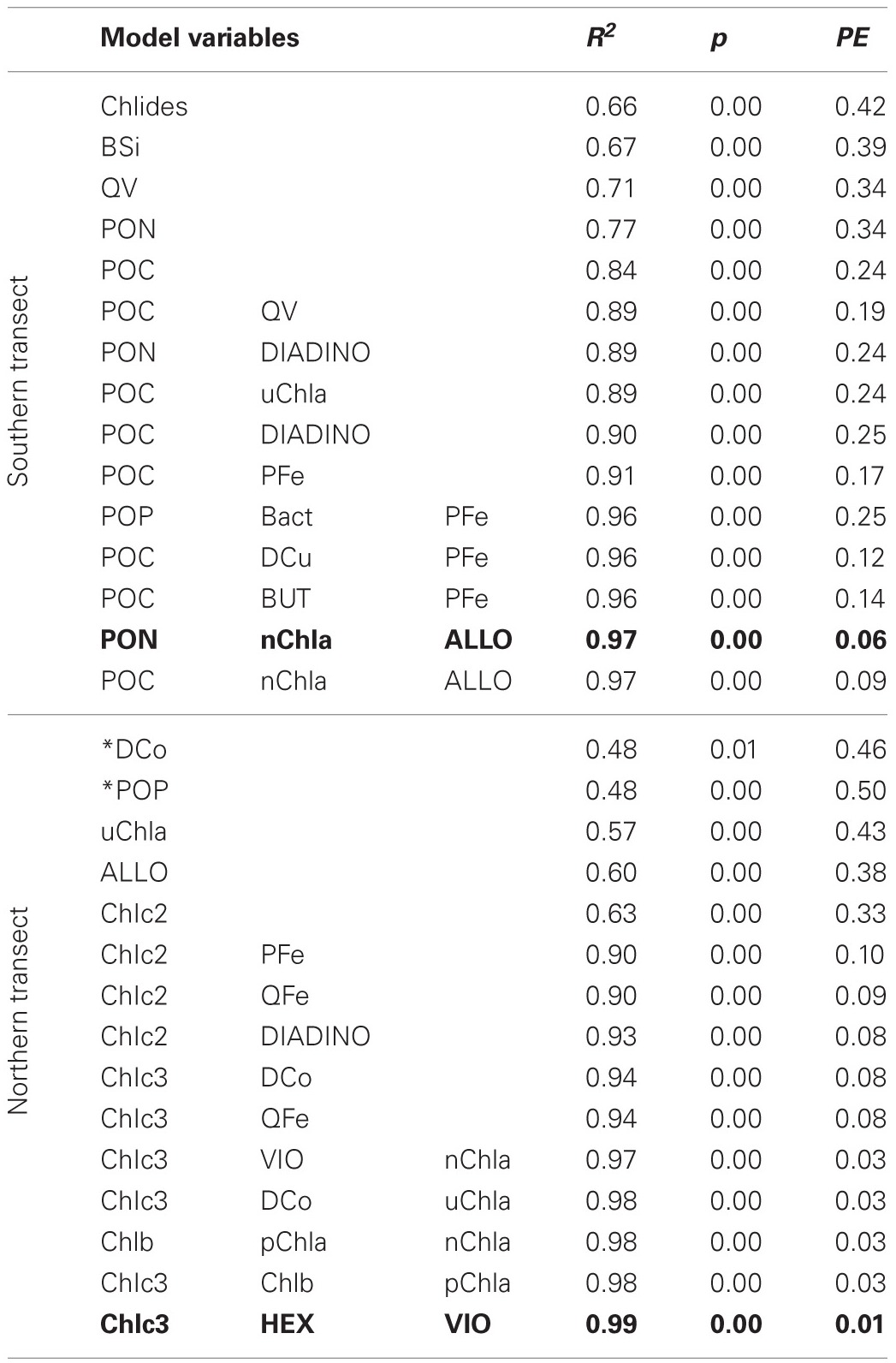
**Linear models and diagnostic statistics for chlorophyll a, only statistically significant regressions for up to three variables are presented**.

Models with the lowest predictive error (PE), determined by leave-one-out cross validation, are bolded. Models produced from hypothesis-testing are marked with an asterisk.

**Table 4:**
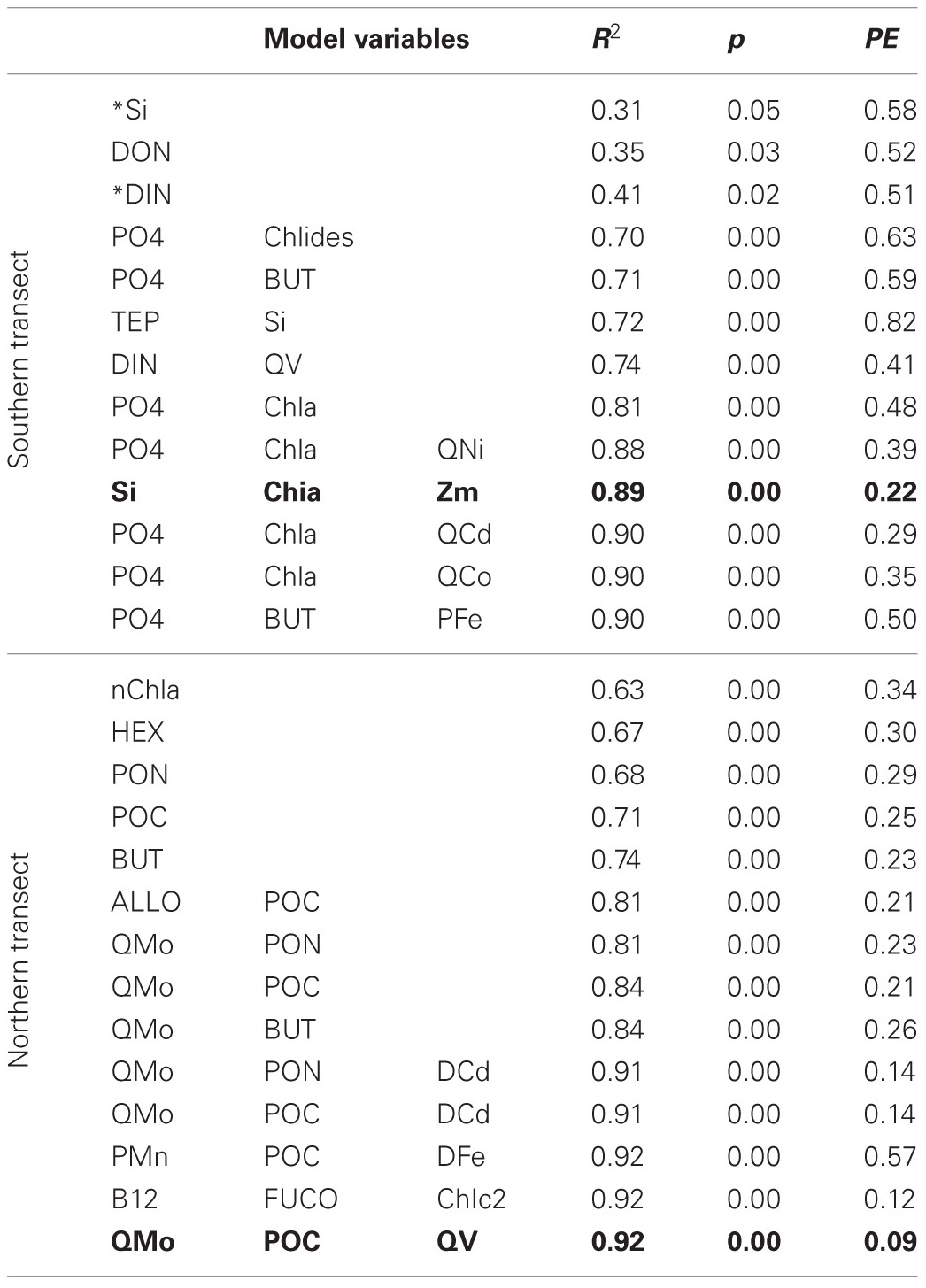
**Linear models and diagnostic statistics for DMS, only statistically significant regressions for up to three variables are presented**.

Models with the lowest predictive error (PE), determined by leave-one-out cross validation, are bolded. Models produced from hypothesis-testing are marked with an asterisk.

**Figure 5:**
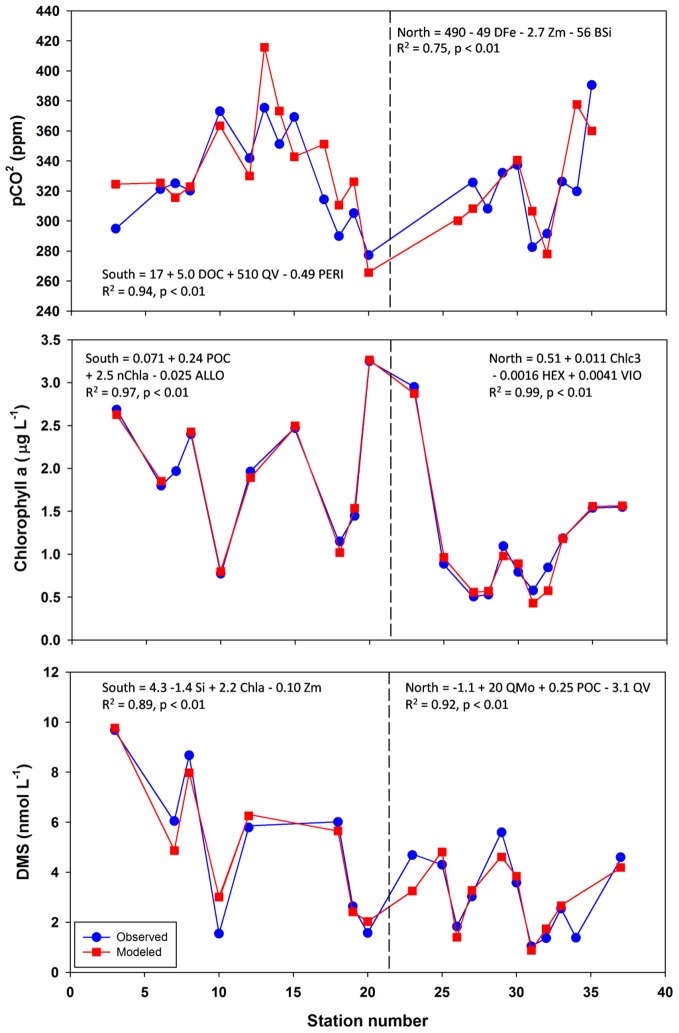
**Observed vs. modeled pCO_2_, chlorophyll a, and DMS along the NASB transect.** Models graphed are those with lowest predictive error as determined by leave-one-out cross-validation. Formulas, R^2^, and *p*-values are given for each regression. Vertical dashed line separates the Southern (left panel, stations 1–23) from Northern transect (right panel, stations 24–37) sections.

Following this, a stepwise linear regression algorithm (Lumley, 2009) was employed to exhaustively calculate polynomial regressions vs. the three response variables for all possible combinations of up to three variables. Statistically significant models were retained and leave-one-out cross-validation performed (Canty and Ripley, 2010) to minimize overfitting. These models and relevant statistical metrics are presented in Tables 2, 3, and 4 with the best-fit model bolded. Best-fit regressions for pCO_2_, DMS, and chlorophyll a for both Northern and Southern transect sections yield good fits and are plotted vs. observed field data in Figure 5. These models and relevant statistical metrics are presented in Tables 2, 3, and 4 with the best-fit model in boldface font.

### pCO_2_ modeling

The best-fit models for pCO_2_ (Table 2, Figure 5) involves DOC (dissolved organic carbon), PERI (peridinin, a pigment characteristic of dinoflagellates), and QV (oxalate-washed P-standardized particulate vanadium concentrations) for the southern transect and DFe (dissolved Fe), Zm (mixed-layer depth), and BSi (biogenic silica) for the northern transect section. For the south, DOC and P-standardized particulate V in particular are present in many of the pCO_2_ regression models. DOC alone yields a statistically significant (*p*-value < 0.05) regression with pCO_2_ with an R^2^ of 0.78. During the 1989 Joint Global Ocean Flux Study experiment in the North Atlantic, depth-integrated DOC was found to be 10× greater than POC (particulate organic carbon), and bacterial production was 30% of total primary production (Lochte et al., 1992). The authors hypothesized that this bacterial production likely metabolized a significant amount of DOC, and this microbial utilization of the DOC pool could explain the inclusion of DOC in the pCO_2_ models. LeBlanc et al. (2009) found dinoflagellates to be a dominant group in the southern portion of the transect, and the fact that dinoflagellates can engage in heterotrophy and osmotrophy (uptake and metabolism of dissolved organic compounds) suggest that they may also contribute to the strong correlation between DOC and pCO_2_ in the southern transect (Burkholder et al., 2008).

**Figure 6:**
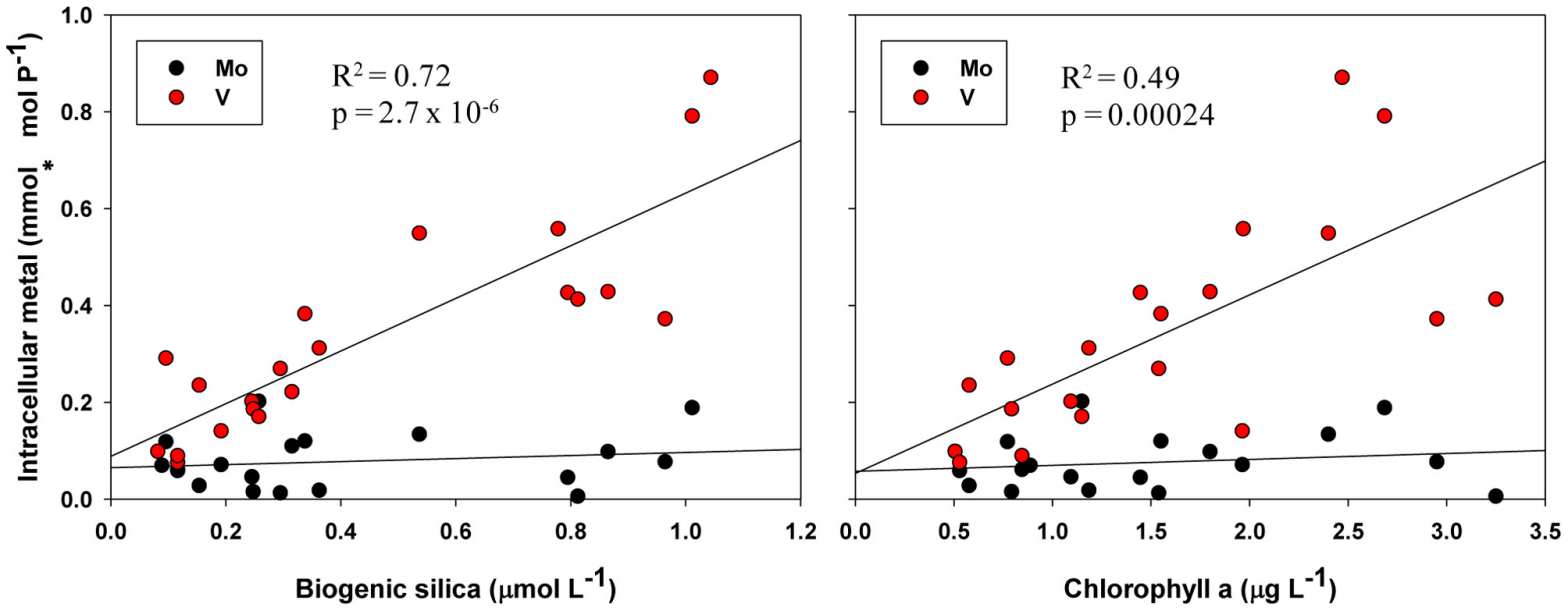
**Oxalate-washed particulate V:P and Mo:P vs. biogenic silica and total chlorophyll a across the entire NASB transect.** *R*^2^ and *p*-values are given for linear regressions of the independent variable vs. intracellular V. Regressions against particulate Mo:P were not statistically significant.

Biological roles for V are not well-characterized, but the inclusion of P-standardized particulate V in many of the best-fit regression models presented here (Tables 2, 3, 4, Figure 5) as well as statistically significant (*p* < 0.05) correlations between oxalate-washed particulate V:P alone and both biogenic silica and chlorophyll a (*R*^2^ = 0.72 and *R*^2^ = 0.49, respectively) across the entire transect (Figure 6) suggests an important relationship. Vanadium and Mo exist chiefly in seawater as oxyanions chemically analogous to PO^3−^_4_ (Crans et al., 2004), so correlations with particulate V:P but not Mo:P (as shown) implies selective uptake of V by the phytoplankton. Tovar-Sanchez and Sañudo-Wilhelmy (2011) and Nuester et al. (2012) both observed high intracellular concentrations of V in sampled *Trichodesmium* colonies (with the latter study finding it to be the most abundant intracellular trace metal), and hypothesized that this enrichment of V might be due to a biochemical role in elimination of reactive oxygen species via V-haloperoxidases and/or passive uptake as an analogue of PO^3−^_4_ during P-limited conditions.

V-containing haloperoxidase activity has been identified in a number of polar and temperate diatoms (Hill and Manley, 2009) and the non-diazotrophic cyanobacterial strain *Synechococcus* CC9311 (Johnson et al., 2011), but the enzyme's function and thus potential relation to pCO_2_ is not well-understood. Since our results indicate both active uptake of V and relate particulate V to photosynthetic biomass variables (chlorophyll a and biogenic silica, Figure 5), they are consistent with an important biological role for this element. However, caution must be taken in inferring causation from correlation models such as those presented here. Further experimentation to verify a direct causal relationship between V and pCO_2_ or algal biomass (such as V-amendments of laboratory cultures or field samples) is necessary before any strong assertions about the role and importance of V can be made.

The best-fit model for pCO_2_ in the north contains variables more typically associated with bloom development (mixed-layer depth, biogenic silica) as well as dissolved Fe, which is likely limiting based on stoichiometric ratios presented here. Regression with likely-limiting dissolved Fe yields a statistically significant regression with an R^2^ of 0.38. The two regressions with VIO (violaxanthin, a pigment characteristic of coccolithophores) have predictive errors much greater than the other models and as such are not considered further here.

### Chlorophyll a modeling

Models for chlorophyll a (Table 3, Figure 5) contain mostly biomass variables in the southern transect section (PON, POC, POP, BSi) and chiefly other pigments in the northern transect section (size-fractionated chlorophyll a, eukaryotic accessory pigments chlorophyll c2 and chlorophyll c3). The best-fit model for the southern transect includes particulate organic nitrogen, the nanophytoplankton fraction of chlorophyll a, and alloxanthin, which is a pigment characteristic of cryptophytes (Roy et al., 2006). Regressions containing oxalate-washed particulate V:P concentrations also occur here, also. The best-fit model for chlorophyll a in the northern transect section includes chlorophyll c3 and 19′hexanoyloxyfucoxanthin (both characteristic of the then-dominant coccolithophores) and violaxanthin, a pigment characteristic of dinoflagellates.

### DMS modeling

DMS linear regression models (Table 4, Figure 5) for the southern transect subset include dissolved inorganic nutrients (phosphate, DIN, Si) as well as chlorophyll a. For the northern section, they involve mostly biomass indicators (PON, POC) and oxalate-washed Mo:P concentrations. The best-fit model for the southern section comprises dissolved silica, chlorophyll a, and mixed-layer depth—all variables associated with the classical NASB progression. For the north, the best-fit model involves oxalate-washed Mo:P and V:P as well as POC. As referenced earlier, Mo is a cofactor in DMSO reductase (Schindelin et al., 1996). Mo is relatively more depleted stoichiometrically (Table 1) in the northern transect section than in the south. This along with the inclusion of particulate Mo:P in many of the DMS regressions for the north suggests that Mo may be important for the production of DMS. No relationship between V and DMS production has been previously suggested in the literature, and a better understanding of the biological role of V is needed to understand the relationship between V and DMS implied by the inclusion of oxalate-washed particulate V:P in the best-fit regression for the northern transect.

## Conclusions

The 2005 NASB data analyzed here indicate, on the basis of nutrient stoichiometry, that the bloom could have been both Si and Fe-limited at the time of sampling, and Co and B_1_ concentrations were also potentially limiting. With the caveat that correlation models do not imply causation, linear regression modeling suggest the importance of mixed-layer depth and dissolved Si and Fe concentrations in relation to pCO_2_ and DMS concentrations. The inclusion of oxalate-washed particulate Mo:P and V:P concentrations alongside parameters traditionally of importance in the NASB (mixed layer depth, dissolved Fe, Si) in the models for DMS and pCO_2_, respectively, hint at unknown and potentially important roles for these lesser-studied trace metals, perhaps particularly in the case of V where biological functions are not well elucidated. Further investigations are needed into the possible linkages between V and phytoplankton biology, and between particulate Mo:P and DMS production in the oceans.

### Conflict of interest statement

The authors declare that the research was conducted in the absence of any commercial or financial relationships that could be construed as a potential conflict of interest.

## Appendix

Variable abbreviations used in linear regression modeling (Tables 2–4, Figure 5).

**Table:**

**Abbreviation**	**Variable**
ALLO	Alloxanthin
B1	Dissolved vitamin B1 (thiamin)
B12	Dissolved vitamin B12 (cobalamin)
Bact	Bacterial abundance
BSi	Biogenic silica
BUT	19′-butanoyloxyfucoxanthin
Chla	Chlorophyll a
Chlb	Chlorophyll b
Chlc2	Chlorophyll c2
Chlc3	Chlorophyll c3
Chlides	Total chlorophyllides
DCd	Dissolved Cd
DCo	Dissolved Co
DCu	Dissolved Cu
DFe	Dissolved Fe
DIADINO	Diadinoxanthin
DIN	Dissolve dinorganic nitrogen
DMo	Dissolved Mo
DMS	Dissolved dimethyl sulfide
DNi	Dissolved Ni
DOC	Dissolved organic carbon
DON	Dissolved organic nitrogen
DV	Dissolved vanadium
DZn	Dissolved zinc
FUCO	Fucoxanthin
HEX	19'Hexanoyloxyfucoxanthin
pChla	pico fraction of chlorophyll a
pCO_2_	Partial pressure of CO_2_
PERI	Peridinin
PFe	Particulate Fe
PIC	Particulate inorganic carbon
PMn	Particulate Mn
PO4	Dissolved ortho-phosphate
POC	Particulate organic carbon
PON	Particulate organic nitrogen
POP	Particulate organic phosphorus
QCd	P-standardized particulate quotas of Cd
QCo	P-standardized particulate quotas of Co
QCu	P-standardized particulate quotas of Cu
QFe	P-standardized particulate quotas of Fe
QMn	P-standardized particulate quotas of Mn
QMo	P-standardized particulate quotas of Mo
QNi	P-standardized particulate quotas of Ni
QV	P-standardized particulate quotas of V
Si	Dissolved silicic acid
TEP	Transparent exopolymer particles
uChla	Micro fraction of chlorophyll a
VIO	Violoaxanthin
ZEA	Zeaxanthin
Zm	Depth of the mixed layer
Zn	Depth of the nitracline
